# Knee and ankle biomechanics during recovery from primary, secondary, and bilateral anterior cruciate ligament injuries: a quasi-experimental study

**DOI:** 10.1590/1516-3180.2024.0497.R1.13072025

**Published:** 2025-12-15

**Authors:** Sérgio Loureiro Nuno, Carlos Romero-Morales, Daniel López-López, Marta Elena Losa-Iglesias, Ricardo Becerro-de-Bengoa-Vallejo, Juan Gómez-Salgado, João Guerra, Miguel Ángel Saavedra-García

**Affiliations:** I Research, Health and Podiatry Group, Department of Health Sciences, Faculty of Nursing and Podiatry, Universidade da Coruña, Ferrol, Spain.; II Full Professor, Department of Physiotherapy, Faculty of Medicine, Health and Sports, Universidad Europea de Madrid, Villaviciosa de Odón, Madrid, Spain.; III Full Professor, Research, Health and Podiatry Group, Department of Health Sciences, Faculty of Nursing and Podiatry, Universidade da Coruña, Ferrol, Spain.; IV Full Professor, Department of Nursing and Stomatology, Faculty of Health Sciences, Universidad Rey Juan Carlos, Madrid, Spain.; V Full Professor, Faculty of Nursing, Physiotherapy and Podiatry, Universidad Complutense de Madrid, Madrid, Spain.; VI Full Professor, Department of Sociology, Social Work and Public Health, Faculty of Labour Sciences, University of Huelva, Huelva, Spain, and Safety and Health Postgraduate Programme, Universidad Espíritu Santo, Guayaquil, Ecuador.; VII Senior Lecturer, Research Unit in Physiotherapy, Cross I&D Lisbon Research Center, Escola Superior de Saúde da Cruz Vermelha Portuguesa, Lisbon, Portugal, and Instituto de Biofísica e Engenharia Biomédica, Faculdade de Ciências, Universidade de Lisboa, Lisbon, Portugal.; VIII Senior Lecturer, Group of Research in Sport Science (INCIDE), Department of Physical Education and Sport, Universidade da Coruña, A Coruña, Spain.

**Keywords:** Anterior cruciate ligament reconstruction, Weight-bearing, Rehabilitation, Y-Balance test, Knee movements, Movement system, Ligament diseases

## Abstract

**BACKGROUND:**

Anterior cruciate ligament (ACL) tears are common knee injuries with a known but vague association with secondary joint injuries. The extent to which these injuries are preventable remains unclear.

**OBJECTIVE:**

This study aimed to assess the functional differences in knee and ankle dorsiflexion biomechanics with full loading on one leg and to understand whether it could be a key point as a progressive method in ACL reconstruction, considering both legs and three different groups.

**DESIGN AND SETTING:**

A quasi-experimental study of Medical Centers in an outpatient clinic in Portugal

**METHODS:**

The Y balance test (YBT) was used to evaluate and analyze the association between ankle dorsiflexion range of motion (DF-ROM) and knee flexion. DF-ROM and knee flexion were used to compare the deficits between the operated and uninvolved limbs in all three groups (ACL-I, ACL-II, and ACL-III).

**RESULTS:**

Ankle DF-ROM and knee flexion assessed during the YBT were associated with higher knee flexion ROM, identifying individuals who were better prepared for the next phase of the guideline. The study results provide preliminary data for future studies that use prospective longitudinal research and involve large patient populations to establish prognostic biomechanical markers for determining long-term dynamic stability after ACL reconstruction.

**CONCLUSIONS:**

In the three groups with a history of ACL injury, compensations and kinematic asymmetries in dorsal flexion and knee flexion were observed in the operated and control legs both at 6 and 8 weeks of treatment.

## INTRODUCTION

 Anterior cruciate ligament (ACL) tears are common knee injury with a known but vague association with secondary joint injuries, and the extent to which these injuries are preventable remains unclear.^1-3^ ACL injury impairs neuromuscular function putting athletes with ACL injury at a high risk of ACL reinjury and functional asymmetries throughout the recovery process.^1-4^ Biomechanical assessment of kinematic changes over time can elucidate responses to neuromuscular intervention and ACL reconstruction (ACLR).^3,5-7^

 Patients with ACLR commonly adopt poor movement patterns that potentially place them at an increased risk of reinjury if left untreated.^8,9^ Persistent intermember biomechanical asymmetries during walking have been reported at the time of return to physical activity.^4,9,10^

 Available ACL rehabilitation protocols in the scientific literature has substantial heterogeneity.^11-19^ Movement compensations following ACLR are commonly observed in patients and can be assessed with various techniques.^20,21^ Asymmetric functional activities mechanics can persist for years after ACLR despite full recovery of strength and clinically assessed function.^19,22 ,23^ Practice guidelines suggest that the increased risk of future ACL injuries may be attributable to changes in neuromuscular function and biomechanics, such as greater internal rotation of the hip and dynamic valgus of the knee.^23-25^ Although asymmetric knee joint mechanics have been associated with the consequent development of post-traumatic osteoarthritis, current clinical interventions have not been successful in fully restoring normal kinematics.^4,25-31^

 Several post-ACLR rehabilitation protocols have been proposed to improve muscle strength and knee stability through muscle-strengthening and joint proprioception exercises. The proposed protocols are mainly based on the biological tissue healing and remodeling timeframes.^23,28^

 With the increase in publications available to rehabilitation specialists, a functional and safe progression for athletes needs to be identified to make progress in their postoperative ACLR rehabilitation program, particularly in joint mobility and weight bearing, which need to be controlled in the postrehabilitation period.^32,34^

 This article presents a progression system between 6 and 8 weeks after ACLR, which can form an important aspect of the movement-based retraining process, providing structural and patient autonomy. Although the incidence of ACL injuries in athletes has increased exponentially, no differentiated concern regarding primary, recurrent, or bilateral injuries has been raised. Monitoring knee and ankle function and movement is crucial to ensure safe transition. Changes in movement patterns during weight-bearing are unclear. Therefore, these factors need to be examined in order to identify strategies for preventing secondary ACL injuries after ACLR. 

 This study presents a novel approach to understanding clinical outcomes, including the ACL, for the first time, and in cases of recurrence. In this study, we investigated this topic and the novelty of the relationship between knee and ankle kinematics at a stage when progressively more body load is applied. We hypothesized that most athletes would not be ready to reach load control in the unipodal tests 6 weeks postoperatively, regardless of whether it was the first knee injury, recurrence, or bilateral injury. Removing crutches prematurely in patients with knee conditions may exacerbate knee kinematic asymmetries; however, their effect remains uncertain. 

## OBJECTIVE

 This study aimed to comprehensively assess deficits in functional movement patterns and dynamic control, as well as side-to-side asymmetries in athletes after ACL, and to compare them with athletes who have suffered a recurrence of ACL injury, as well as with athletes who have suffered bilateral ACL injury. 

## METHODS

### Design and sample

 This study was approved by the Bioethics Committee and registered under number NCT06050005 at clinicaltrials.gov. Furthermore, the Consolidated Statement for Reporting Trials (CONSORT) statement and checklists were considered.^35^ All procedures followed the principles of the Declaration of Helsinki for Good Clinical Practice and were approved by our Institutional Ethics Committee (Process CE.CSJD/P1.23). The Ethics Committee for Health provided favorable opinions on the implementation of the doctoral project and showed itself available provided support to achieve the proposed research objectives. 

 This was a three-group quasi-experimental study. Participants were recruited from medical services. The same examiner conducted all tests, and all participants completed the informed consent form. 

 The testing was performed in a sports medicine laboratory. The sample size was calculated based on pilot data and previous literature.^8,36 ,37^ With 11 participants in each group, we had 80% power (α = 0.05) to detect a clinically relevant 50% improvement in knee and ankle-dorsiflexion biomechanics during functional tests. A priori power analysis calculations were based on sagittal plane knee biomechanics and indicated that 36 athletes were needed to detect a medium effect size (0.3) with β = 0.20 and α = 0.05. 

 A total of 88 volunteers were selected to participate in the study and divided into three groups. 

 Eighty-eight active participants were identified to participate in this research study and met the inclusion criteria. 

 The ACL-I group (25.8 ± 9.1 years old) of both sexes (15 women and 29 men) corresponds to the group of individuals who had an ACL injury for the first time. The ACL-II group includes individuals who have re-injured the ACL in the same knee (27.1 ± 7.5 years old) of both sexes (9 women and 16 men). According to this clinical criterion, injury to the same knee will occur after at least 1 year. The ACL-III group includes individuals who had ACL injury bilaterally (30.4 ± 6.8 years old) of both sexes (five females and fourteen males), considering the current injury for the study, but the contralateral leg in the past had the same rehabilitation. 

 The athletes were aged between 18 and 45 years (median, 12.7 years), participated in sports for > 50 h a year before their first ACL injury, and wanted to return to their pre-injury activity levels. ACLR was performed by the same medical team of experienced orthopedic surgeons, and the athletes participated in postoperative rehabilitation at the same physiotherapy clinic. Strict enrolment criteria were applied to ensure a homogeneous entry level. 

 Participants were selected after obtaining informed consent and involved specific predefined inclusion and non-inclusion criteria as described below. The participants were nonsmokers, exercised regularly, monitored by a coach/teacher, and did not take any dietary supplements or medications. 

 The inclusion criteria were as follows: began physiotherapy in the preoperative context and continuation of recovery up to 2 weeks postoperatively; age ≥ 18 years; and participants with previous severe chondral defects were not included in the study, but meniscus repair or meniscectomy performed at the time of ACLR was tolerated. 

 The exclusion criteria were concomitant bilateral injury with several chondral defects/history of surgery or contralateral dysfunction; meniscal suture; cartilaginous injury; injury to the medial collateral ligament, lateral collateral ligament, and posterior cruciate ligament; concomitant intra- and extra-articular plastic surgery; complex injury from any accident; complex tibial condyle fracture; rheumatoid arthritis; recent heart disease; intermittent claudication; neuropathies; and cognitive alterations. 

### Procedure

 The eligible participants underwent an initial assessment. All the follow-up evaluations were performed by the same evaluator. The participants’ age, sex, body mass index (BMI), injury history, ACL rehabilitation (i.e., self-reported duration), surgical details (i.e., self-reported graft type and meniscal procedures), and previous activity level were obtained at baseline. 

**Figure 1:**
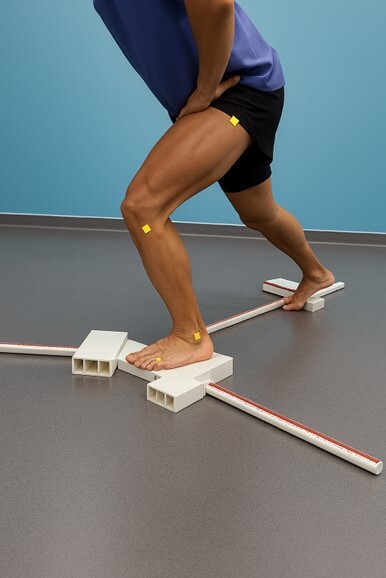
Experimental setup and bone references for range of motion calculations. Participants stand at the center of the equipment and push a plastic piece to the anterior, posteromedial, and posterolateral directions.

 For screening, each potential participant’s non-weight-bearing dorsiflexion range of motion (DF-ROM) was assessed for both legs, with the participant lying supine on a treatment table. The examiner moved the ankle into plantar flexion and placed it in a subtalar-neutral position by palpation. They passively dorsiflexed the ankle while maintaining a subtalar-neutral position until the point of first resistance. Then, the examiner measured the angle formed by the shaft of the fibula and the lateral midline of the foot using a standard goniometer. This assessment was performed with the subtalar joint in a neutral position to avoid movement compensation at the subtalar and midtarsal joints, and to effectively evaluate talocrural joint motion. Subsequently, the participants were guided to perform knee flexion. If associated pain was present at any time, the ROM count was finalized. After the examiner recorded the ROM measurements, the participants were prepared for motion analysis data collection using the YBT (**Figure 1**). 

 The evaluation period was divided into two phases: phase I, 6 weeks postoperatively, and phase II, 8 weeks postoperatively. Each phase presents unique clinical considerations and challenges. The ACLR treatment in all athletes began on postoperative day 1. All individuals were provided as much weight as possible to tolerate pain with crutches on postoperative day 1. On the postoperative day 21 (3 ), the amount of weight bearing increased. Full weight-bearing began in phase I (6 weeks) and was progressively achieved until phase II (8 weeks). The kinematic analysis focused on the operated leg. 

### Statistical analysis

 Statistical analyses were performed using the Prism software (GraphPad Software Inc. Version 10.2.0, Boston, Massachusetts, United States). Data were transferred to the IBM SPSS Statistics 27.0.01.0 package for Windows (Armonk, New York, United States) program and statistically evaluated. Before proceeding with the statistical analysis, controls were made regarding the absence of data entry errors and whether the parameters were within the expected range. 

 The outcome variables analyzed included the DF-ROM, knee flexion range of motion (ROM), and dynamic balance (anterior reach, posteromedial reach, and posterolateral reach). Noncategorical baseline and outcome data were tested for normal distribution using the Kolmogorov–Smirnov test. Results are expressed as frequencies or as means ± standard deviations (SDs). Group demographics were compared using a one-way analysis of variance (ANOVA). To determine differences between the three groups and time (phase I — pretest and phase II — posttest), a 3 × 2 repeated measures ANOVA was conducted, followed by post hoc comparison. The within-group factor (pretest to posttest) as the main effect of time and the between-group factor as the main effect of group were considered. 

## RESULTS

 A total of 88 patients participated in functional performance tests. Forty-four patients who underwent primary ACL were initially included in this study (ACL — I group). A total of 25 patients were included in the ACL repair group with recurrent lesions (ACL — II group). The remaining 19 patients had bilateral ACL lesions that underwent reconstruction (ACL III group). **Table 1** presents demographic data. For the preoperative program, all participants were physically active, defined as 30 min of physical activity at least three times per week before surgery. The time from injury to surgery ranged from 2 weeks to 2 months (mean = 29.1 days, SD = 17.3, median = 33.5), and was significantly different between the groups (P < 0.001). 

**Table 1:** Basic data of the population studied

	**ACL I group**	**ACL II group**	**ACL III group**	**P value**
Age (years)	25.8 ± 9.1	27.1 ± 7.5	30.4 ± 6.9	0.173
Weight (kg)	74.0 ± 15.1	79.4 ± 18.0	68.0 ± 8.1	0.192
Height (cm)	176.0 ± 18.0	172.9 ± 14.8	171.0 ± 11.0	0.620
BMI (kg/m^2^)	23.1 ± 2.8	24.8 ± 2.0	22.9 ± 1.8	0.551
Years of training before injury	11.1 ± 4.8	14.6 ± 5.9	17.5 ± 7.0	0.082

ACL = anterior cruciate ligament; BMI = body mass index.

 At baseline, no significant differences (P > 0.05) in the demographic data was observed among the three intervention groups (**Table 1**): age ([P = 0.173), mass (P = 0.192), height (P = 0.620), BMI (P = 0.551), and years of training before injury (P = 0.082]). The same was valid between the groups at baseline for any of the dependent variables of interest (P < 0.05), indicating that the groups were comparable in terms of their initial anthropometry. 

 The ankle DF-ROM and knee flexion for each group, along with means and SDs, are represented and related to the distance traveled during the YBT and are presented in **Table 2** for the ACLR leg and **Table 3** for the control leg. 

**Table 2:** Means and standard deviations of dorsal flexion and knee flexion in the Y balance test

**Variables ACLR leg**	**Group**	**Phase I** Pre-test 6 weeks postoperatively **Mean ± SD**	**Phase II** Post-test 8 weeks postoperatively **Mean ± SD**	**P value**		
				**Main effect of time**	**Main effect of group**	**Group × time interaction**
DF-ROM ANTYBT	**ACL-I group**	40.7 ± 17.9	47.7 ± 12.3	F = 140.142 P < 0.001^*^	F = 43.233 P < 0.001^*^	F = 60.609 P < 0.001^*^
	**ACL-II group**	43.4 ± 10.1	45.4 ± 15.2			
	**ACL-III group**	18.1 ± 19.0	39.1 ± 13.8			
DF-ROM POSTLYBT	**ACL-I group**	24.0 ± 12.5	33.3 ± 14.0	F = 99.117 P < 0.001^*^	F = 20.487 P < 0.001^*^	F = 36.928 P < 0.001^*^
	**ACL-II group**	37.1 ± 17.0	38.2 ± 19.8			
	**ACL-III group**	30.1 ± 8.8	32.3 ± 8.8			
DF-ROM POSTMYBT	**ACL-I group**	32.8 ± 13.9	31.0 ± 9.5	F = 16.191 P < 0.001^*^	F = 2.987 P < 0.085	F = 8.222 P < 0.001^*^
	**ACL-II group**	27.7 ± 8.8	28.2 ± 9.6			
	**ACL-III group**	33.1 ± 17.6	35.1 ± 17.3			
KF-ROM ANTYBT	**ACL-I group**	51.8 ± 17.2	52.8 ± 20.3	F = 109.007 P < 0.001^*^	F = 38.411 P < 0.001^*^	F = 440.571 P < 0.001^*^
	**ACL-II group**	41.8 ± 20.3	50.1 ± 14.4			
	**ACL-III group**	40.8 ± 21.2	42.1 ± 8.9			
KF-ROM POSTLYBT	**ACL-I group**	48.7 ± 30.1	59.0 ± 22.5	F = 75.120 P < 0.001^*^	F = 19.007 P < 0.001^*^	F = 23.007 P < 0.001^*^
	**ACL-II group**	58.8 ± 28.5	58.7 ± 13.1			
	**ACL-III group**	61.8 ± 7.7	62.2 ± 7.2			
KF-ROM POSTMYBT	**ACL-I group**	58.8 ± 14.4	64.9 ± 14.4	F = 28.142 P < 0.001^*^	F = 3.930 P < 0.062	F = 10.531 P < 0.001^*^
	**ACL-II group**	57.4 ± 14.8	63.7 ± 24.8			
	**ACL-III group**	57.5 ± 12.3	59.5 ± 10.3			

Values refer to the anterior cruciate ligament reconstructed leg; P < 0.05 indicates a statistically significant difference. ACL = anterior cruciate ligament; ACLR = anterior cruciate ligament reconstruction; SD = standard deviation; ROM = range of motion; DF = dorsiflexion; KF = knee flexion; ANTYBT = anterior movement in Y balance test; POSMYBT = posteromedial movement in Y balance test; POSTLYBT = posterolateral movement in Y balance test.

**Table 3:** Means and standard deviations of dorsal flexion and knee flexion in the Y balance test

**Variables Control leg**	**Group**	**Phase I** Pretest 6 weeks postoperatively **Mean ± SD**	**Phase II** Posttest 8 weeks postoperatively **Mean ± SD**	**P value**		
				**Main effect of time**	**Main effect of group**	**Group × time interaction**
DF-ROM ANTYBT	**ACL-I group**	45.8 ± 11.4	48.6 ± 11.9	F = 74.553 P < 0.001^*^	F = 6.852 P < 0.004^*^	F = 14.248 P < 0.001^*^
	**ACL-II group**	46.4 ± 9.0	50.3 ± 17.7			
	**ACL-III group**	40.6 ± 16.8	41.0 ± 17.9			
DF-ROM POSTLYBT	**ACL-I group**	38.4 ± 9.3	35.9 ± 12.3	F = 10.232 P < 0.012	F = 14.032 P < 0.002^*^	F = 5.102 P < 0.0035^*^
	**ACL-II group**	40.6 ± 10.1	40.4 ± 15.2			
	**ACL-III group**	35.7 ± 15.3	35.7 ± 18.6			
DF-ROM POSTMYBT	**ACL-I group**	33.9 ± 10.6	37.6 ± 14.5	F = 42.743 P < 0.001^*^	F = 14.553 P < 0.001^*^	F = 8.383 P < 0.001^*^
	**ACL-II group**	43.8 ± 14.1	33.4 ± 14.2			
	**ACL-III group**	37.5 ± 17.0	37.7 ± 19.0			
KF-ROM ANTYBT	**ACL-I group**	52.7 ± 15.6	56.7 ± 18.1	F = 82.111 P < 0.001^*^	F = 43.288 P < 0.001^*^	F = 45.431 P < 0.001^*^
	**ACL-II group**	45.8 ± 10.8	53.7 ± 18.8			
	**ACL-III group**	39.7 ± 27.9	41.7 ± 13.9			
KF-ROM POSTLYBT	**ACL-I group**	54.8 ± 18.1	60.8 ± 10.3	F = 94.077 P < 0.001^*^	F = 10.084 P < 0.0047^*^	F = 12.248 P < 0.001^*^
	**ACL-II group**	59.4 ± 15.9	60.4 ± 7.9			
	**ACL-III group**	52.0 ± 17.9	63.0 ± 14.2			
KF-ROM POSTMYBT	**ACL-I group**	60.4 ± 14.8	65.7 ± 17.2	F = 102.825 P < 0.001^*^	F = 32.003 P < 0.001^*^	F = 37.561 P < 0.001^*^
	**ACL-II group**	53.9 ± 20.0	64.4 ± 13.1			
	**ACL-III group**	52.3 ± 17.0	60.7 ± 18.0			

The control values in groups I and II refer to the non-operated legs. Group III refers to legs with a history of anterior cruciate ligament injury; P < 0.05, statistically significant difference. ACL = anterior cruciate ligament; SD = standard deviation; ROM = range of motion; DF = dorsiflexion; KF = knee flexion; ANTYBT = anterior movement in Y balance test; POSMYBT = posteromedial movement in Y balance test; POSTLYBT = posterolateral movement in Y balance test

 Significant group × time interaction effects, main effect of time, and main effect of group were observed in all directions (P < 0.05). No significant between-group differences in the posteromedial direction of DF-ROM and knee flexion (ACLR leg) were observed. No significant effect of time on the DF-ROM in the posterolateral direction (control leg) was noted. 

 At anterior for DF-ROM and knee flexion at ACLR leg (P = 0.001), posterolateral for DF-ROM and knee flexion at ACLR leg (P = 0.001) and posteromedial for differences between phases and group × time interaction significant differences were found between ACL-I and other groups. 

 Additionally, there were significant differences in the anterior, posteromedial, and posterolateral directions for the DF-ROM and knee flexion for leg control. 

## DISCUSSION

 This study assessed the functional differences with full loading on one leg and aimed to elucidate whether it could be a key factor as a progressive method in ACLR, considering the control leg and the difference in values between the legs and three groups. 

 Among the athletes in this study, the ACL-I group (n = 44) showed the best kinematic results for both dorsal ankle and knee flexion. At 6 weeks of assessment (phase I), the individuals were not yet ready for unipodal loading, and statistically significant differences were observed between the groups and control leg. The most commonly used criterion for progression in clinical practice is time from surgery, which is derived from the biological healing time. Meanwhile, injury and rehabilitation of a ligament results in a drastic change in its structure and physiology and creates a situation where ligament function is restored by the formation of scar tissue that is biologically and biomechanically inferior to the tissue it replaces.^18,38,39^ These findings imply that most participants in our study group were not eligible for unipodal functional exercise. Despite satisfactory results between phases I and II, functional asymmetries still existed between the operated and control legs. Symmetrical exercise is a prerequisite for walking, running, and pivoting sports.^29,40 ,41^ Anticipating recovery timings can be a risk factor for re-injury and can contribute to functional asymmetry at the end of recovery 

 Full extension and weight bearing were achieved on the first postoperative day. Early postoperative assessments were performed at 6 and 8 weeks. The hallmark of this phase is enabling athletes to perform basic functional activities until they can tolerate more advanced activities. We consider this one of the positive effects of early rehabilitation and is consistent with the results of other studies.^42-44^ Early accelerated rehabilitation characterized by joint mobilization and weight-bearing within 3 days postoperatively should be the mainstream approach in isolated ACL surgeries. When concomitant injuries (meniscal and cartilage) are present, the early rehabilitation phase should be adapted according to medical instructions.^18,34^

 Based on this premise, a recent study has reported a compensatory pattern of decreased knee flexion and increased hip flexion angles in athletes after ACLR.^45^ All of which could increase the risk of ACL injury. The reason for the reduction in knee flexion angle and dorsal flexion of the ankle remains unclear.^46-49^ Some studies have suggested that altered activation or reduced strength of the quadriceps results in a decreased ability to flex the knee during demanding single-leg tasks, whereas others have suggested that the strength of the hamstrings can be reduced.^6,50^

 In our study, the change in knee and ankle ROM was statistically significant, which, according to theory, is more protective against injury to the knee joint (i.e., increased flexion); however, we did not report patient outcomes or minimal clinically important differences.^12,16,51^ Increasing plantar flexor extensibility and dorsiflexion ROM can help reduce the load on the ACL.^52,53^

 Information regarding risk factors for ACL injury vary in the current literature.^51,53 ,54^ Athletes after ACLR with asymmetric ROM in knee and ankle DF may be related to incorrect long-term recovery after ACLR, and loading timings must always be framed.^21 ,26,27^

 In our study, patients who followed a partial-load regimen for up to 6 weeks postoperatively and progressively performed full-load exercises to be fully loaded at 8 weeks had better results. Deficits in amplitude and movement after an injury are well known. However, how these deficits correlate or can be predictors of the stages of evolution or lead to a new injury requires further exploration. 

 Interventions in a clinical population emphasize the difficulty of balancing the safety of athletes and testing to capture a representative population. Therefore, we implemented strict clinical criteria for study participation. This may also have biased our study cohort by selecting athletes who functioned at a higher baseline level. Adherence in our study was the fact that the athlete completed the 8 weeks of treatment and the pre/post biomechanical testing sessions in order to be included. 

 Asymmetries in the lower limbs may suggest an increased risk of ACL injury during these maneuvers, and suggesting the implementation of more position-specific ACL prevention programs may be reasonable, as the more dynamic movements associated with central players may develop specific muscles differently than those of wing players. 

 However, this study has some limitations. First, this study did not test the electromyographic signal characteristics of the patients’ electromyographic signals, and strong conclusions were drawn regarding the pattern of muscle activity in patients with ACLR. Second, we investigated interventions in the general population of athletes, which could be a mistake because of the differences between sports. Lastly, patients with limb dominance were excluded from this study. 

## CONCLUSIONS

 In the three groups with a history of ACL injury, compensations and kinematic asymmetries in dorsal flexion and knee flexion were noted in the operated and control legs both at 6 and 8 weeks of treatment. 

 Asymmetric performance on the YBT was considered valid for predicting the evaluation and control of the risk of lower limb injuries. This study provides new information on how clinical measurements of the DF-ROM and knee flexion are associated with biomechanical variables suggested as risk factors for ACL injury. Functional testing should be performed before treatment progression. 

## Data Availability

The data that support the findings of this study are available from the corresponding author, Daniel López López, upon reasonable request.
